# Pancreatic adenocarcinoma third line systemic treatments: a retrospective cohort study

**DOI:** 10.1186/s12885-024-12016-z

**Published:** 2024-02-26

**Authors:** A. Gueiderikh, A. Tarabay, M. Abdelouahab, C. Smolenschi, M. L. Tanguy, M. Valery, D. Malka, T. Pudlarz, A. Fuerea, V. Boige, A. Hollebecque, M. Ducreux, A. Boilève

**Affiliations:** 1 Département de médecine oncologique, Gustave Roussy, 94800 Villejuif, France; 2 Université Paris Saclay, 91471 Orsay, France; 3 Département de statistiques, Gustave Roussy, 94800 Villejuif, France; 4 Gustave Roussy, DITEP, 94800 Villejuif, France; 5 Département d’oncologie médicale, Institut Mutualiste Montsouris, 75014 Paris, France

**Keywords:** Pancreatic adenocarcinoma, mPDAC, Erlotinib, Gemcitabine, FOLFIRINOX

## Abstract

**Background:**

Chemotherapy for metastatic pancreatic adenocarcinoma (PDAC) primarily relies on FOLFIRINOX (LV5FU- irinotecan – Oxaliplatine) and Gemcitabine – Nab-Paclitaxel in the first-line setting. However, second-lines remain less well-defined and there is limited data regarding third-line treatments. The objective of our study was to determine the proportion of patients advancing to third line chemotherapy, to outline the various third-line chemotherapy regimens used in routine practice and to evaluate their respective efficacy.

**Methods:**

A retrospective single-center cohort from 2010-2022 compiled baseline characteristics, treatment outcomes and survival of PDAC patients who received at least one chemotherapy line in a French tertiary-center. Overall survivals (OS) were analyzed using a Cox multivariable model.

**Results:**

In total, 676 patients were included, with a median follow-up time of 69.4 months, (Interquartile Range (IQR) = 72.1). Of these, 251 patients (37%) that proceeded to 3^rd^-line chemotherapy. The median PFS in 3^rd^ line was 2.03 months, [CI95%: 1.83, 2.36]. The median 3^rd^ line overall survival was 5.5 months, [CI95%: 4.8, 6.3]. In multivariable analysis erlotinib-based chemotherapy was found to be deleterious (HR=2.38, [CI95%: 1.30, 4.34], *p*=0.005) compared to fluoropyrimidine-based chemotherapy in terms of 3^rd^ line overall survival while gemcitabine monotherapy showed a tendency towards negative outcomes. First and 2^nd^ line chemotherapies sequence didn’t influence 3^rd^ line outcome.

**Conclusion:**

In our cohort, one-third of treated patients proceeded to 3^rd^ line chemotherapy resulting in a 5.5 months median 3^rd^ line OS, consistent with treatments at advanced stage. Our results argue against the use of erlotinib and gemcitabine monotherapy.

**Supplementary Information:**

The online version contains supplementary material available at 10.1186/s12885-024-12016-z.

## Introduction

Pancreatic cancer (PDAC) is the fouth leading cause of cancer mortality in men and women in Europe [1] and the seventh worldwide [2]. The incidence is increasing over the last decades in the USA [3], Europe [4] and in France [5] thus marking it a major public health concern. Identified risk factors include age [3], smoking, obesity, genetics, diabetes, diet and inactivity [6]. Early onset pancreatic cancer are defined by cases arising before 50 years and is associated with a poor prognosis [7]. Low survival rates, not exceeding 10% at 5 years, underscore the importance of research dedicated to chemotherapy improvement.

To date, systemic treatments primarily rely on chemotherapeutics and targeted therapies or immune-oncology drugs are used to a lesser extent. The first-line chemotherapy regimen had long relied on gemcitabine treatment. However, in 2000s, the combination of gemcitabine and erlotinib [8] or gemcitabine and capecitabine [9] demonstrated a modest but statistically significant overall survival (OS) increase. Subsequently, two main phase III trials established the current standard-of care regimens. On the first hand, FOLFIRINOX (LV5FU- Irinotecan – Oxaliplatine) [10] showed a survival increase but also had associated increased toxicity. Further adaptations of the protocol improved its tolerance without altering its efficacy [11, 12]. On the other hand, gemcitabine and albumin–bound paclitaxel (Nab-paclitaxel) proved more beneficial over gemcitabine alone [13]. Although no study directly compared the two treatments, FOLFIRINOX outperformed gemcitabine/Nab-paclitaxel in some meta-analysis or retrospective data [14, 15] while other studies indicated similar efficacy for both regimens but favored gemcitabine-Nab-paclitaxel due to its better tolerance [16, 17]. Gemcitabine combinations with oxaliplatin, capecitabine or other drugs also demonstrated potential advantages over gemcitabine monotherapy in retrospective studies [18, 19]. Recently, NALIRIFOX (Nal-Iri (liposomal irinotecan)–LV5FU- oxaliplatin) showed superiority over gemcitabine/nab-paclitaxel and may be considered as a first-line option pending its reimbursment [20]. Furthermore, there is no direct comparison of NALIRIFOX and FOLFIRINOX and the median OS were similar in their respective trials.

In the second-line chemotherapy, switching from one first-line regimen to another is viable due to their non-overlapping toxicity profiles as assessed in retrospective studies [21]. An estimated 20 to 50% of patients might benefit from this strategy [15]. Moreover, the Nal-Iri–5FU regimen, specifically developed in this setting [22], showed an OS benefit over 5-FU alone for patients who had previously undergone gemcitabine-based chemotherapy. Currently, no regimen has demonstrated superiority in the second-line and data are lacking for a definitive third-line therapy [23].

We conducted a retrospective cohort study that included all consecutive patients treated for a metastatic PDAC from 2010 to 2022 in our tertiary-center hospital. The aim of our study was to determine the proportion of patients advancing to third-line chemotherapy, to outline the different 3^rd^ line chemotherapy regimens used in everyday practice and to evaluate their respective efficacy.

### Patients and methods

This study details a retrospective cohort conducted in a French tertiary-center hospital. The eligibility criteria included: patients with histologically-proven PDAC, age of over 18, and those who received at least one chemotherapy regimen, treated in our institution between 2010 and 2022. Study data were collected and managed using *REDCap* electronic data capture tools hosted at Gustave Roussy.

The primary objectives were to assess the proportion of patients undergoing 3^rd^ line chemotherapy and to compare the efficacy of various 3^rd^ line chemotherapy regimens based on 3^rd^ line overall survival. The secondary objectives included determining the progression-free survival of 3^rd^ line chemotherapy and identifying the optimal sequence between first and second-line chemotherapies to advance to 3^rd^ line regarding overall survival from 1^st^ line chemotherapy initiation.

Chemotherapy regimens were categorized into a 5-class categorical variable. Gemcitabine monotherapy was singled out as a separate regimen due to its proven inferiority compared to other regimens in the litterature. Fluoropyrimidine-based regimens covered FOLFOX (LV5FU – oxaliplatin), FOLFIRI (LV5FU – irinotecan), FOLFIRINOX, modified FOLFIRINOX, XELOX (capecitabine - oxaliplatin)/ XELIRI (capecitabine – irinotecan), capecitabine and LV5FU (5 Fluoro-Uracile). Gemcitabine combination regimens included gemcitabine – paclitaxel, gemcitabine – Nab-paclitaxel, gemcitabine–oxaliplatine, and alternating gemcitabine and 5FU treatments. Erlotinib-based chemotherapies comprised erlotinib alone, erlotinib-gemcitabine, erlotinib-capecitabine. The “Other chemotherapies” group encompassed PARP inhibitors, bidirectional chemotherapy, gemcitabine-capecitabine, LV5FU–carboplatine, docetaxel, weekly paclitaxel, clinical trial drugs and other less common treatments labeled as “various chemotherapy” group. The term “Various chemotherapy” group referred to regimens administered fewer than 4 times in the cohort and is detailed in Table S1. When one of the described drugs was given as a control in a clinical trial, it was re-categorized to its corresponding category.

For 1^st^ and 2^nd^ line chemotherapy sequence evaluation in the multivariable analysis, 1^st^ and 2^nd^ line chemotherapy sequences were arranged as: (i) L1= fluoropyrimidine based regimen and L2= gemcitabine combination regimen; (ii) L1= gemcitabine combination and L2= fluoropyrimidine based regimen, (iii) L1= gemcitabine monotherapy and L2=fluoropyrimidine based regimen; (iv) Other combination of chemotherapies. For detailed 1^st^ and 2^nd^ line chemotherapy sequence evaluation, two other sequences were added: (v) L1= fluoropyrimidine based regimen and L2= Gemcitabine monotherapy and (vi): L1= fluoropyrimidine based regimen and L2= fluoropyrimidine based regimen.

Several chemotherapy sequences were compared head-to-head with time from 2nd line chemotherapy initiation to the time to 3^rd^ line initiation or to death analyzed in a competing risk survival model taking death as a competing event.

### Statistics

The median follow-up and associated IQR were estimated using the reversed Kaplan-Meier method.

OS and PFS in 3rd line were calculated from the start of 3rd line therapy initiation and estimated with the Kaplan-Meier method. Patients without an event at the date of last follow-up were censored at this date. The 95% bilateral confidence intervals of OS and PFS were calculated using Greenwood formula. To identify factors that might influence overall survival in the 3rd line, we used a multivariable Cox regression model.

A Fine-Gray model assessed the impact of the second line therapy on the probability of proceeding to 3rd line. The time to 3rd line therapy was calculated from the start of 2nd line therapy. Patients alive at the date of last follow-up without having received a 3rd line therapy were censored. Death before initiating a 3rd line therapy was treated as a competing risk.

No imputation of missing data was undertaken. Analysis was restricted to subjects with complete data on the variable involved in the analysis and the number of missing subjects is indicated for each table.

All tests were two-sided. A *p*-value ≤ 0.05 indicated statistically significant.

The R software was used for all statistical analyses*.*

## Results

**Fig 1:**
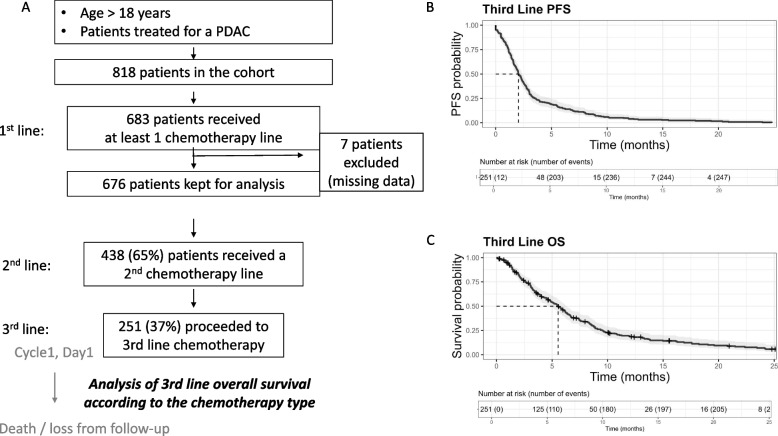
**A** Flowchart of the study, **B** Third line Progression free survival, **C** Third line Overall Survival

Out of the 818 consecutive PDAC patients included in the database, 683 presented a good enough general status to benefit from at least one chemotherapy line and 7 patients were excluded due to missing data. In total, 676 patients were included in the study (Fig. 1). The median follow-up time for the whole population was 69.5 months (IQR= 72.1). Among them, 251 patients (37%) received at least 3 systemic treatments lines. The median follow-up from 3^rd^ line therapy initiation onwards was 25.1 months (IQR=37.7). The median PFS in 3^rd^ line was 2.03 months ([CI95%: 1.83, 2.36]) and median 3^rd^ line chemotherapy overall survival was 5.5 months ([CI95%: 4.8, 6.3]) (Fig. 1B and C). Main characteristics of the whole population of patients who proceeded to 3^rd^ line chemotherapy are depicted in Table 1. Fifty-two percent of patients were male. There were few patients aged 70 years or over (34 patients (14%)) or aged under 50 years (43 patients (17%)) (Table 1). One hundred and sixty-one patients (96%) had a 0-1 performance status at diagnosis (Table 1). One hundred fifty-five patients (62%) were metastatic at diagnosis, including 115 patients (46%) presenting liver metastases. Forty-eight patients (28%) reported a regular alcohol intake and 68 (39%) were smokers. Fifty-five patients (40%) presented a venous thrombosis during disease evolution (Table 1). Molecular characteristics of tumors, when tested, are presented in Supplementary Table S2. Detailed characteristics of the disease at diagnosis are presented in Supplementary Table S3. Sixty-three patients benefited from former local treatments, including 25 patients undergoing a surgery, 30 - radiotherapy and 7 – interventional radiology for metastasis.

**Table 1:** Population description

	Patients (%) (Total=251 patients)
**Gender**	
- Man	130 (52%)
- Woman	121 (48%)
**Age**	
- a) under 50	43 (17%)
- b) 50 to 70	174 (69%)
- c) 70 or more	34 (14%)
**Performance status at diagnosis**	
- 0 - 1	161 (96%)
- 2 and more	6 (4%)
- Missing	84
**Disease stage at diagnosis**	
- Non Metastatic	96 (38%)
- Metastatic	155 (62%)
**Liver metastasis**	
- No	136 (54%)
- Yes	115 (46%)
**Regular alcohol intake**	
- No	122 (72%)
- Yes	48 (28%)
- Missing	81
**Smoking status**	
- No	106 (61%)
- Yes	68 (39%)
- Missing	77
**Associated thrombosis**	
- No	83 (60%)
- Yes	55 (40%)
- Missing	113

**Fig 2:**
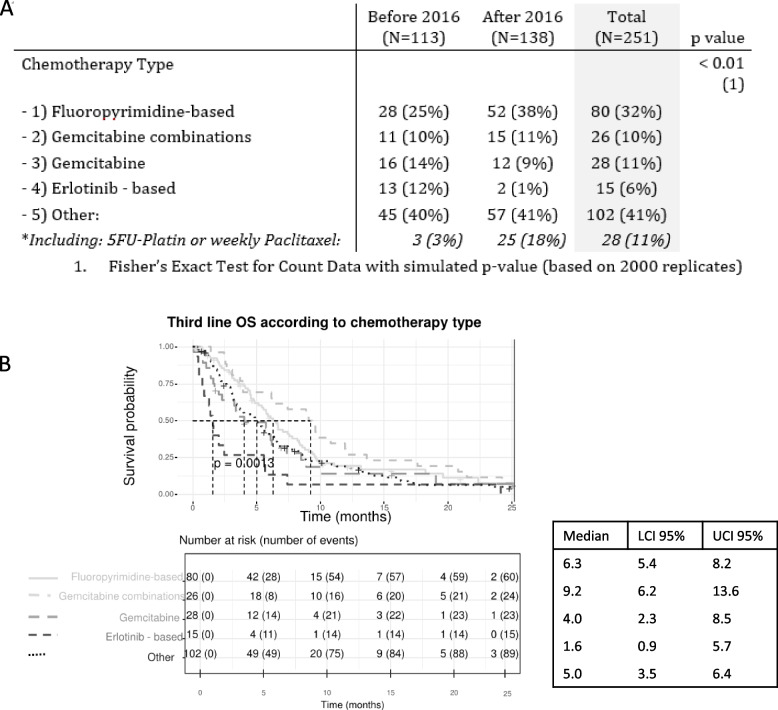
**A** Chemotherapy type evolution over years, **B** Survival probability of different chemotherapy groups and associated median survival using a Kaplan Meier analysis

To fulfill the description of patients that proceeded to 3rd line chemotherapy, we described the chemotherapies they received. The chemotherapy regimens administered in 3^rd^ line were very diverse, with more than 20 different regimens identified (Supplementary Table S4). Chemotherapy type distribution was different among chemotherapy lines. First line chemotherapy was mainly fluoropyrimidine – based (71%) and 2^nd^ line chemotherapy was mainly gemcitabine (28%) or gemcitabine combination (21%) based. Third-line chemotherapy was distributed as follows: 80 patients (32%) received fluoropyrimidine-based chemotherapy, 26 patients (10%) received gemcitabine combination chemotherapy, 28 patients (11%) received gemcitabine monotherapy, 15 (6%) received erlotinib and 102 patients (41%) received other chemotherapy regimens (among which 49 (20%) received a drug tested in a clinical trial) (Fig. 2A and Supplementary Table S4 ).

The pattern of prescription evolution over time is presented in Fig. 2A. After 2016, erlotinib-based regimen and gemcitabine monotherapy prescription declined in 3^rd^ line, contrary to fluoropyrimidine- based chemotherapy or 5FU-platin and weekly paclitaxel regimens (Table 2).

**Table 2:** Factors influencing 3rd line overall survival (multivariable Cox model)

**Characteristic**	**HR**^a^	**CI 95%**^a^	***p*-value**
**3**^**rd**^** line chemotherapy type**			
1) Fluoropyrimidine-based	—	—	
2) Gemcitabine combinations	0.74	0.45, 1.21	0.2
3) Gemcitabine	1.63	0.98, 2.69	0.058
4) Erlotinib - based	2.38	1.30, 4.34	**0.005**
5) Other	1.20	0.85, 1.68	0.3
**1**^**st**^** and 2**^**nd**^** line chemotherapy sequence**			
L1=Gem and L2=FU-based	—	—	
L1=FU-based and L2=Gem combinations	1.19	0.61, 2.32	0.6
L1=Gem combinations and L2 =FU-based	0.96	0.45, 2.03	>0.9
Other sequence	1.06	0.59, 1.91	0.8
**Age at diagnosis**			
a) under 50	—	—	
b) 50 to 70	0.94	0.64, 1.39	0.8
c) 70 or more	1.00	0.59, 1.70	>0.9
**Year of treatment**			
Before 2016	—	—	
After 2016	1.04	0.77, 1.41	0.8
**Reason for 1st line chemotherapy arrest**			
Progression	—	—	
Other	0.58	0.34, 0.99	**0.045**
Toxicity	0.27	0.08, 0.90	**0.033**
**Reason for 2nd line chemotherapy arrest**			
Progression	—	—	
Other	0.77	0.27, 2.21	0.6
Toxicity	0.82	0.44, 1.53	0.5
**Hepatic metastases at diagnosis**			
No	—	—	
Yes	1.58	1.18, 2.12	**0.002**

^a^*HR* Hazard Ratio, *CI* Confidence Interval, *n*=248; 3 patients excluded for missing data on cause of 1st and 2nd line chemotherapies arrest

Among patients that proceeded to 3^rd^ line chemotherapy, cause for 1^st^ and 2^nd^ line chemotherapy arrest was mainly progression, but some patients arrested for toxicity or other reasons (including intensification, temporally loss from follow-up, intent of a curative treatment or therapeutic pause). Overall response rate (ORR) to 1^st^ line chemotherapy was 73% with partial response in 34% of patients and stability in 37% of patients and ORR in 2^nd^ line was 53% with mainly stability (45% of patients) (Supplementary Table S5).

Focusing on the efficacy of various 3^rd^ line chemotherapy regimens based on focusing on 3^rd^ line chemotherapy survival, Kaplan Meier survival curves seem to suggest that patients receiving erlotinib-based chemotherapy had a worse prognosis than others receiving 3^rd^ line treatments (Fig. 2B). Then, using a multivariable Cox survival model adjusted for age at diagnosis, year of diagnosis, reason for 1^st^ and 2^nd^ line chemotherapy arrest, presence of liver metastases and the sequence of drugs received in 1^st^ and 2^nd^ line, we confirmed that receiving erlotinib-based chemotherapy (HR=2.38, [CI95%: 1.30, 4.34], *p*=0.005) led to a worse outcome than receiving a fluoropyrimidine based chemotherapy in 3^rd^ line. The presence of liver metastases (HR= 1.58, [CI95% : 1.18, 2.12], *p*=0.002) were also associated with a poor 3^rd^ line overall survival whereas 1^st^ line chemotherapy arrest for toxicity (HR=0.27, [CI95%: 0.08, 0.90], *p*=0.033) or other cause (HR=0,58, [CI95%: 0.54, 0.99], *p*=0.045) was associated with a better 3^rd^ line overall survival. Receiving gemcitabine chemotherapy regimen tended to be deleterious without reaching statistical significance (Table 2). Sequence of chemotherapies received in 1^st^ and 2^nd^ line did not influence 3^rd^ line overall survival.

As cause for 1^st^ line chemotherapy arrest influences 3^rd^ line chemotherapy outcomes, we aimed at describing patients who ended first-line treatment without progression. 8 patients (3%) stopped 1^st^ line chemotherapy for toxicity and 24 (10%) stopped for other reason, including intensification, intent of a curative treatment, loss from follow-up or therapeutic pause (Supplementary Table S5). We found that those patients mainly received a FOLFIRINOX chemotherapy in 1^st^ line and benefited from a reintroduction of an adapted fluoropydimidine-based chemotherapy (containing only one or 2 molecules) in 2^nd^ and in 3^rd^ line (Supplementary Table S6). On the contrary in the global population only 32% of patients received a fluoropyrimidine-based regimen in 2^nd^ line (Supplementary Table S4). Time to 3^rd^ line initiation or to death or to loss from follow-up was 10.4 months in this population ([CI95%: 8.95-NA]) when it was 8.9 months in the global population ([CI95%: 7.2-10.2]).

We then investigated more deeply whether 1^st^ and 2^nd^ line chemotherapies sequences impacted on the probability to proceed to 3^rd^ line and on the delay to 3^rd^ line initiation (Fig. S1 A). At the beginning of 2^nd^ and 3^rd^ line, patients presented similar repartitions of 1^st^ and 2^nd^ line chemotherapy sequences (Fig. S1 B). The probability of proceeding to 3^rd^ line treatment for patients who received a fluoropyrimidine based chemotherapy in first and in second line was similar to patients who received a fluoropyrimidine based chemotherapy in first line and a gemcitabine combination chemotherapy in second line (Fig. S1 C left panel).

Eventually, focusing on fluoropyrimidine-based and gemcitabine combination regimen, we studied whether initiating treatment with one or the other of the regimen influenced the probability to proceeded to 3^rd^ line after 2^nd^ line initiation in our dataset. Comparison of both sequences head-to-head did not reveal neither difference regarding the probability to proceed to 3^rd^ line (Fig. S1 C right panel).

## Discussion

This work represents the largest study published up-to-date evaluating the role of third line chemotherapy treatment in metastatic PDAC. With the improvement of first and second treatment lines more patients present a good performance status at second line progression and the best chemotherapy choice in 3^rd^ line is a daily concern. In this retrospective cohort study, we report a median 3^rd^ line survival was 5.5 months with many types of chemotherapy regimen received, due to lack of treatments recommendation. Chemotherapy regimen based on erlotinib in 3^rd^ line and liver metastases at diagnosis were associated with a poor outcome. Importantly, those results were obtained after adjustment for year of diagnosis, as we showed that in our institution erlotinib-based treatment were less prescribed over time. Gemcitabine monotherapy also tended to unfavorable outcome in 3^rd^ line. On the other hand, we could not find any difference in 1^st^ and 2^nd^ line chemotherapy sequence regarding the two main prescribed regimen: fluoropyrimidine-based or gemcitabine combination regimens, neither regarding OS, nor the proportion of patients proceeding to 3^rd^ line.

Our study confirms the pejorative value of liver metastases in pancreatic cancer, as only 46% of 3^rd^ line proceeding patients presented liver metastases at diagnosis and it remained a factor of poor outcome in 3^rd^ line. On the contrary, 1^st^ line arrest without progression was associated with a good outcome. Duration of 1^st^ and 2nd line was similar in those patients and in the global population, meaning that it was not a population in which 3^rd^ line was given earlier, which would have accounted for a better outcome. This group represents mainly patients who arrested first line FOLFIRINOX for toxicity, need for a therapeutic pause or intent of intensification, and who benefited from an adapted fluoropyrimidine regimen in second line. To understand whether this chemotherapy choice was responsible for the good outcome of those patients, we performed a competitive survival analysis in the global population of patients beginning 2^nd^ line and we obtained similar probability of proceeding to 3^rd^ line with the use of this chemotherapy sequence compared to the mainly used fluoropyrimidine – gemcitabine combination sequence. Thus, good responders to fluoropyrimidine treatment in 1^st^ line mainly benefit from its reintroduction in 2^nd^ and 3^rd^ line but this strategy can’t be recommended in the global population.

Accordingly with previous articles, our multivariable models were adjusted for age [15], year of diagnosis, liver metastases [13, 22, 15] , reason for 1^st^ and 2^nd^ line chemotherapy arrest and the sequence of drugs received in 1^st^ and 2^nd^ line. Other stratification factors were used in literature: performance status [10, 13, 22, 24], primary tumor localization (head vs body or tail) [10], albumin [22], time since receiving most recent anticancer therapy [22], tumor stage at diagnosis [22], baseline CA19-9 [15, 22, 24] number of metastatic sites [15], peritoneal carcinomatosis [15], CRP>5mg/dL [24]. Also, diabetes has been described to be associated with a worse outcome of chemotherapy treatment in pancreatic cancer [25]. We decided not include Performance status, diabetes and CA19.9 at diagnosis in our analysis due to an excess of missing data. Adding gender, localization in pancreas head or neck, visualization on CT scan, number of metastatic sites or metastatic stage at diagnosis did not change the results of our multivariable model. Mutations profiling could not be included in multivariable analyses due to excess of missing data (only half of the patients benefited from a molecular profiling). Diabetes was still less frequent in patients proceeding to 3^rd^ line chemotherapy in accordance with former studies [25].

Interestingly, even if young onset pancreatic cancer is described to have poorer prognosis [7], we didn’t find it in our cohort regarding 3^rd^ line issue. Moreover, elderly patients have usually worse outcome over chemotherapy treatment [26] or present more toxicities [27] and are often excluded from phase III trials (ie. in Conroy et. al [10] age over 76 years was an exclusion criterion). We still did not find an influence of age on the issue of 3^rd^ line chemotherapy. Comorbidities could not be evaluated as not-oncologic medical history was not assessed in our cohort.

The vast diversity of chemotherapies prescribed in our cohort reflects the lack of current consensus regarding PDAC chemotherapy, and especially the lack of standard treatments in 3^rd^ line. We had to perform a grouping of several classes for the analysis in a way that reflected practice evolution over time and which preserved sufficient effectives. Capecitabine – gemcitabine regimen was prescribed in only three patients in 3^rd^ line and appears in the group “other”. Only two patients benefited from Nal-IRI-5FU in our cohort and this regimen is part of the “Various chemotherapy” group (Table S11). PARP inhibitors were not prescribed in 3^rd^ line. All contents of the chemotherapies that remained classified as “Various chemotherapy” besides the 22 other initial chemotherapy categories can be found in Supplementary Table 1. “Other chemotherapy type” included many clinical trial drugs, whose modest outcome probably reflect the difficulties of pancreatic cancer drug development already noted by other authors [23].Taking into account those limitation, we didn’t find any difference between 1^st^ line fluroropyrimidine based chemotherapy then 2^nd^ line gemcitabine combination chemotherapy and the reverse sequence in terms of delay up to proceeding to 3^rd^ line.

Chemotherapy re-challenge (the use of the exactly same chemotherapy type in 1^st^ and 2^nd^ line) was present in only 21/684 patients and was thus neglected (considered as a new chemotherapy line) but all the patients that were recorded receiving a new chemotherapy line were considered as progressing or changing treatment strategy after the previous line. Supplementary Table S6 shows rather a change in prescription from FOLFIRINOX in 1^st^ line to adapted regimen in 3rd line (containing only one or 2 molecules). This prescription is performed in our center when 1^st^ line FOLFIRINOX lead to a response and when progression is not observed immediately.

We acknowledge several limitations of our study. First, data were monocentric and collected in a tertiary-referral center, whose practice may differ from a general hospital. The retrospective design is also a manifest constraint, despite the need for real world data in this situation. Efficacy of Folfirinox and Gemcitabine-Nab paclitaxel in 1st/2nd line were analyzed retrospectively, but this important question deserves a randomized trial to be assessed without bias. Also, no multiplicity correction was performed in the analysis, and some of the outlined differences could be false-positive results. Those monocentric retrospective data deserve external validation in additional studies. Then, the amount of missing data for some criteria (due to its retrospective design) impeded us to analyze their impact in the multivariable analysis and the grouping of different chemotherapy regimen to get large enough effectives may have conditioned the results. Also, the combination of locally advanced and metastatic at diagnosis patients meant that 1st line chemotherapy may be neoadjuvant for the first ones. However, this fact was taken into account in the adjustment factors of the multivariable model.

In conclusion, a quite large proportion of patients that receive 1^st^ line chemotherapy proceeded to 3^rd^ line chemotherapy (37%) in our tertiary-referral center. Fluoropyrimidine-based, gemcitabine and paclitaxel-based or other regimen as 5FU-carbolatin or weekly paclitaxel regimen perform equally in 3^rd^ line, whereas erlotinib-based chemotherapy was associated with a poor survival. In regard of our results, it seems appropriate to continue to disfavor erlotinib-based treatments in 3^rd^ line chemotherapy, as it was done in our center over years. Gemcitabine monotherapy also tended to be deleterious, as it was already shown for first line treatment. 1^st^ and 2^nd^ line chemotherapies did not influence the issue of 3^rd^ line but in good responders to 1^st^ line FOLFIRINOX or those who changed chemotherapy line for toxicity, reintroduction of a fluoropyrimidine based regimen in 2^nd^ and 3^rd^ line is a good option.

### Supplementary Information

**Supplementary Material 1.**

**Supplementary Material 2.**

**Supplementary Material 3.**

**Supplementary Material 4.**

**Supplementary Material 5.**

**Supplementary Material 6.**

## Data Availability

Data analyzed in this manuscript is part of a retrospective cohort. Data access should be directed to alice.boileve@gustaveroussy.fr.

## Abbreviations

PDAC: Pancreatic Adenocarcinoma
OS: Overall Survival
PFS: Progression Free Survival
LV5FU: 5 Fluoro-Uracile
FOLFIRINOX: LV5FU- Irinotecan Oxaliplatine
FOLFOX: LV5FU oxaliplatin
FOLFIRI: LV5FU irinotecan
NALIRIFOX: Nal-Iri (liposomal irinotecan)–LV5FU- oxaliplatin
XELOX: Capecitabine oxaliplatin
XELIRI: Capecitabine irinotecan
