# Supplementary material for: Pancreatic adenocarcinoma third line systemic treatments: a retrospective cohort study
Source: BMC Cancer. 2024 Feb 26;24:272. doi: 10.1186/s12885-024-12016-z (PMC10898186; doi:10.1186/s12885-024-12016-z)

A

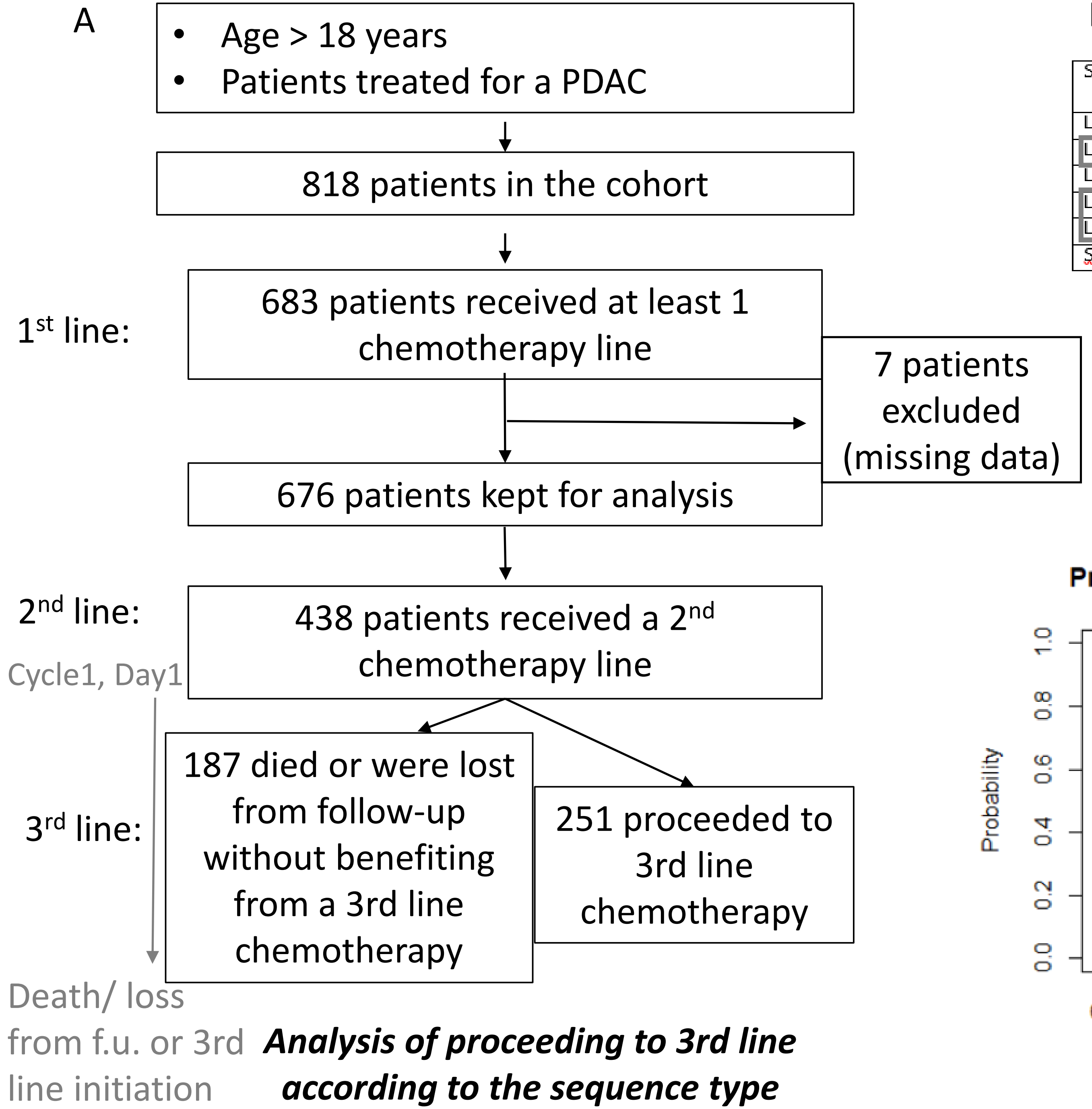

B

Sequence	2nd line (total=438) N (%)	3rd line (total=251) N (%)	Proportion of patients in 2nd line proceeding to 3rd line
L1=FU based and L2=FU based	69 (16%)	47 (19%)	68%
L1=FU based and L2=Gem	118 (27%)	58 (23%)	49%
L1=FU based and L2=Gem combi	86 (20%)	48 (19%)	56%
L1=Gem combi and L2 =FU based	29 (7%)	21 (8%)	72%
L1=Gem and L2= FU based	33 (8%)	15 (6%)	45%
Sequence=Other	103(24%)	62 (25%)	60%

C

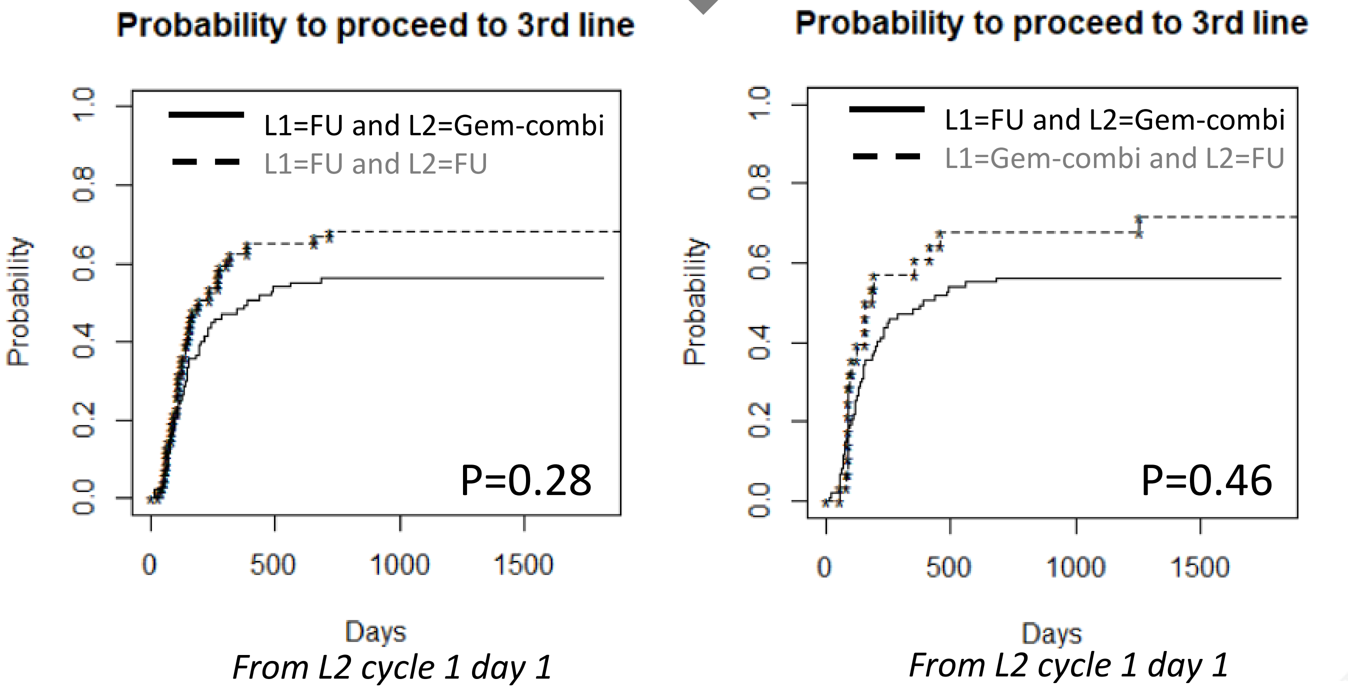

Supplement: Supplementary file 1 — Supplementary Material 1. [file 12885_2024_12016_MOESM1_ESM.pdf]
