# Supplementary material for: Pancreatic adenocarcinoma third line systemic treatments: a retrospective cohort study
Source: BMC Cancer. 2024 Feb 26;24:272. doi: 10.1186/s12885-024-12016-z (PMC10898186; doi:10.1186/s12885-024-12016-z)
Supplement: Supplementary file 2 — Supplementary Material 2. [file 12885_2024_12016_MOESM2_ESM.doc]

**Supplementary Table S1: “Various chemotherapies” group description**

|  | **Chemotherapy** | **Number of occurrences** |
| --- | --- | --- |
| **1** | 5-FU + Nal-IRI | 2 |
| **2** | Anti-MEK | 3 |
| **3** | CAPECITABINE + IRINOTECAN | 1 |
| **4** | CAPECITABINE + MITOMYCINE | 2 |
| **5** | CAPECITABINE + OXALIPLATINE | 3 |
| **6** | CAPECITABINE + TRASTUZUMAB | 1 |
| **7** | Carboplatine | 1 |
| **8** | CARBOPLATINE + 5FU | 1 |
| **9** | CISPLATINE + ETOPOSIDE | 1 |
| **10** | DOCETAXEL + CAPECITABINE | 1 |
| **11** | DOCETAXEL + CISPLATINE | 1 |
| **12** | EPIRUBICINE | 1 |
| **13** | GEMCITABINE + BEVACIZUMAB | 1 |
| **14** | GEMCITABINE + CARBOPLATINE | 2 |
| **15** | GEMCITABINE + CISPLATINE | 2 |
| **16** | GEMCITABINE + MITOMYCINE | 1 |
| **17** | GEMCITABINE + Nab-PACLITAXEL + PEMBROLIZUMAB | 1 |
| **18** | GEMCITABINE + PACLITAXEL + CAPECITABINE | 1 |
| **19** | Gemcitabine carboplatine | 1 |
| **20** | Irinotecan | 2 |
| **21** | LV5FU2 + CARBOPLATINE then OLAPARIB maintenance | 1 |
| **22** | LV5FU2 + MITOMYCINE | 1 |
| **23** | LV5FU2 + Nab-PACLITAXEL | 1 |
| **24** | Nal-IRI | 1 |
| **25** | NIVOLUMAB | 1 |
| **26** | OXALIPLATINE + IRINOTECAN | 1 |
| **27** | PACLITAXEL + CARBOPLATINE | 3 |
| **28** | PACLITAXEL + TRASTUZUMAB | 1 |
| **29** | PCALITAXEL + CARBOPLATINE + PEMBROLIZUMAB | 1 |
| **30** | Pembrolizumab | 2 |
| **31** | weekly PACLITAXEL then Nab-PACLITAXEL | 1 |
