# Supplementary material for: Pancreatic adenocarcinoma third line systemic treatments: a retrospective cohort study
Source: BMC Cancer. 2024 Feb 26;24:272. doi: 10.1186/s12885-024-12016-z (PMC10898186; doi:10.1186/s12885-024-12016-z)
Supplement: Supplementary file 3 — Supplementary Material 3. [file 12885_2024_12016_MOESM3_ESM.doc]

**Supplementary Table S2: Detailed Population Characteristics**

|  | 3 or more Chemotherapy Lines (N=251) |
| --- | --- |
| Diabetes |  |
| - No | 96 (72%) |
| - Yes, after pancreatic cancer diagnosis | 13 (10%) |
| - Yes, before pancreatic cancer diagnosis | 11 (8%) |
| - Yes, concomitant to pancreatic cancer diagnosis | 14 (10%) |
| - Missing | 117 |
| Weight (kg) |  |
| - Median (Range) | 68.0 (42.0, 92.0) |
| - Missing | 166 |
| Family history of cancer |  |
| - No | 212 (84%) |
| - Yes | 39 (16%) |
| - Missing | 0 |
| Number of metastatic sites |  |
| - Median (Range) | 1.0 (0.0, 5.0) |
| Genetic predisposition syndrome |  |
| - No | 12 (71%) |
| - Yes | 5 (29%) |
| - Missing | 234 |
| Somatic genetic testing performed |  |
| No | 123 (49%) |
| Yes | 128 (51%) |
| *KRAS* mutation | 112 (88%) |
| *TP53* mutation | 93 (73%) |
| *CDKN2A* mutation | 35 (27%) |
| *SMAD4* mutation | 12 (9%) |
| *BRCA1/2* mutation | 9 (7%) |
| *ARID1A* mutation | 8 (6%) |
| *SMARCA* mutation | 3 (2%) |
| *ATM* mutation | 5 (4%) |
| MSI Status |  |
| - Yes | 1 (2%) |
| - No | 56 (98%) |
| - Missing | 194 |
| TMB load |  |
| - Median (Range) | 1.3 (0.0, 13.8) |
| - Missing | 208 |
