# Supplementary material for: Pancreatic adenocarcinoma third line systemic treatments: a retrospective cohort study
Source: BMC Cancer. 2024 Feb 26;24:272. doi: 10.1186/s12885-024-12016-z (PMC10898186; doi:10.1186/s12885-024-12016-z)
Supplement: Supplementary file 4 — Supplementary Material 4. [file 12885_2024_12016_MOESM4_ESM.docx]

**Supplementary Table S3: Disease characteristics**

|  | 3 or more Chemotherapy Lines (N=251) |
| --- | --- |
| Dimensions of the lesion (mm) |  |
| - Median (Range) | 34.0 (10.0, 95.0) |
| - Missing | 118 |
| Resequability at diagnosis |  |
| - Borderline | 15 (17%) |
| - Locally advanced | 23 (26%) |
| - Resectable | 50 (57%) |
| - Missing | 163 |
| Localization inside the pancreas: |  |
| - neck | 10 (4%) |
| - head | 104 (41%) |
| - uncinate process | 14 (6%) |
| - body | 57 (23%) |
| - tail | 50 (20%) |
| CT-scan visualization |  |
| - No | 27 (11%) |
| - Yes | 224 (89%) |
| Biliary duct stenosis |  |
| - No | 110 (62%) |
| - Yes | 66 (38%) |
| - Missing | 75 |
| Wirsung duct stenosis |  |
| - No | 80 (51%) |
| - Yes | 77 (49%) |
| - Missing | 94 |
| Number of biliary stents during disease course |  |
| - 0 | 127 (72%) |
| - 1 | 42 (24%) |
| - 2 | 8 (5%) |
| - Missing | 74 |
| EBUS visualization |  |
| - No | 140 (56%) |
| - Yes | 111 (44%) |
| ACE at diagnosis |  |
| - Median (Range) | 23.9 (3.0, 3575.0) |
| - Missing | 201 |
| CA 19.9 at diagnosis |  |
| - Median (Range) | 1300.0 (5.0, 630360.0) |
| - Missing | 144 |
| LDH at diagnosis |  |
| - Median (Range) | 195.0 (122.0, 615.0) |
| - Missing | 216 |
| Number of metastatic sites |  |
| - 0 | 96 (38%) |
| - 1 or 2 | 137 (55%) |
| - 3 or more | 18 (7%) |
| Metastatic site: |  |
| - Liver | 115 (46%) |
| Number of liver metastases |  |
| - 1 | 11 (12%) |
| - 2 | 8 (9%) |
| - 3 | 4 (4%) |
| - 4 or more | 68 (75%) |
| - Missing | 24 |
| - Lung | 33 (13%) |
| - Adrenal gland | 5 (2%) |
| - Peritoneum | 33 (13%) |
| - Bone | 5 (2%) |
| - Lymph node | 26 (10%) |
| - Ovary | 9 (4%) |
| - Other metastatic site | 3 (1%) |
