# Supplementary material for: Pancreatic adenocarcinoma third line systemic treatments: a retrospective cohort study
Source: BMC Cancer. 2024 Feb 26;24:272. doi: 10.1186/s12885-024-12016-z (PMC10898186; doi:10.1186/s12885-024-12016-z)
Supplement: Supplementary file 5 — Supplementary Material 5. [file 12885_2024_12016_MOESM5_ESM.doc]

**Supplementary Table S4: Chemotherapy description by line**

| **Chemotherapy line number:** | **1**  **(N=676)** | **2**  **(N=438)** | **3**  **(N=251)** | **≥ 4 (N=156)** | **Total (N=1521)** |
| --- | --- | --- | --- | --- | --- |
| **Chemotherapy :** |  |  |  |  |  |
| - Alternating Gemcitabine and 5-FU based regimen | 11 (2%) | 0 (0%) | 0 (0%) | 0 (0%) | 11 (1%) |
| - Bidirectional chemotherapy | 0 (0%) | 3 (1%) | 5 (2%) | 2 (1%) | 10 (1%) |
| - Capecitabine | 3 (0%) | 7 (2%) | 3 (1%) | 3 (2%) | 16 (1%) |
| - Clinical Trial | 20 (3%) | 42 (10%) | 49 (20%) | 32 (21%) | 143 (9%) |
| - Docetaxel | 0 (0%) | 2 (0%) | 3 (1%) | 0 (0%) | 5 (0%) |
| - Erlotinib | 0 (0%) | 0 (0%) | 0 (0%) | 1 (1%) | 1 (0%) |
| - Erlotinib + Capecitabine | 1 (0%) | 1 (0%) | 9 (4%) | 7 (4%) | 18 (1%) |
| - Erlotinib + Gemcitabine | 3 (0%) | 3 (1%) | 6 (2%) | 0 (0%) | 12 (1%) |
| - FOLFIRI | 21 (3%) | 46 (11%) | 34 (14%) | 15 (10%) | 116 (8%) |
| - FOLFIRINOX | 352 (52%) | 21 (5%) | 9 (4%) | 1 (1%) | 383 (25%) |
| - FOLFOX | 51 (8%) | 58 (13%) | 30 (12%) | 18 (12%) | 157 (10%) |
| - Gemcitabine | 109 (16%) | 123 (28%) | 28 (11%) | 4 (3%) | 264 (17%) |
| - Gemcitabine + Capecitabine | 0 (0%) | 2 (0%) | 3 (1%) | 4 (3%) | 9 (1%) |
| - Gemcitabine + Nab-Paclitaxel | 33 (5%) | 70 (16%) | 18 (7%) | 13 (8%) | 134 (9%) |
| - Gemcitabine + Oxaliplatine | 10 (1%) | 7 (2%) | 1 (0%) | 3 (2%) | 21 (1%) |
| - Gemcitabine + Paclitaxel | 5 (1%) | 15 (3%) | 7 (3%) | 4 (3%) | 31 (2%) |
| - LV5FU | 1 (0%) | 0 (0%) | 3 (1%) | 2 (1%) | 6 (0%) |
| - LV5FU + Platine | 1 (0%) | 7 (2%) | 13 (5%) | 14 (9%) | 35 (2%) |
| - modified FOLFIRINOX | 52 (8%) | 5 (1%) | 1 (0%) | 1 (1%) | 59 (4%) |
| - PARP inhibitor | 0 (0%) | 4 (1%) | 0 (0%) | 3 (2%) | 7 (0%) |
| - Various chemotherapy | 3 (0%) | 13 (3%) | 14 (6%) | 13 (8%) | 43 (3%) |
| - weekly Paclitaxel | 0 (0%) | 6 (1%) | 15 (6%) | 15 (10%) | 36 (2%) |
| - XELOX/XELIRI | 0 (0%) | 3 (1%) | 0 (0%) | 1 (1%) | 4 (0%) |
| **Chemotherapy Type :** |  |  |  |  |  |
| - 1) Fluoropyrimidine-based | 480 (71%) | 140 (32%) | 80 (32%) | 41 (26%) | 741 (49%) |
| - 2) Gemcitabine combinations | 59 (9%) | 92 (21%) | 26 (10%) | 20 (13%) | 197 (13%) |
| - 3) Gemcitabine | 109 (16%) | 123 (28%) | 28 (11%) | 4 (3%) | 264 (17%) |
| - 4) Erlotinib - based | 4 (1%) | 4 (1%) | 15 (6%) | 8 (5%) | 31 (2%) |
| - 5) Other | 24 (4%) | 79 (18%) | 102 (41%) | 83 (53%) | 288 (19%) |
