# Supplementary material for: Pancreatic adenocarcinoma third line systemic treatments: a retrospective cohort study
Source: BMC Cancer. 2024 Feb 26;24:272. doi: 10.1186/s12885-024-12016-z (PMC10898186; doi:10.1186/s12885-024-12016-z)
Supplement: Supplementary file 6 — Supplementary Material 6. [file 12885_2024_12016_MOESM6_ESM.docx]

**Supplementary Table S5: First and second chemotherapy lines description**

|  | 3 or more Chemotherapy Lines (N=251) |
| --- | --- |
| Reason for 1st line chemotherapy arrest |  |
| - Other | 24 (10%) |
| - Progression | 218 (87%) |
| - Toxicity | 8 (3%) |
| - Missing | 1 |
| Best response to 1st line chemotherapy |  |
| - Complete response | 4 (2%) |
| - Partial response | 76 (34%) |
| - Progression | 61 (27%) |
| - Stabiliy | 83 (37%) |
| - Missing | 27 |
| Chemotherapy Type |  |
| - 1) Fluoropyrimidine-based | 190 (76%) |
| - 2) Gemcitabine combinations | 26 (10%) |
| - 3) Gemcitabine | 22 (9%) |
| - 4) Erlotinib - based | 3 (1%) |
| - 5) Other | 10 (4%) |
| Reason for 2nd line chemotherapy arrest |  |
| - Other | 8 (3%) |
| - Progression | 226 (91%) |
| - Toxicity | 14 (6%) |
| - Missing | 3 |
| Best response to 2nd line chemotherapy |  |
| - Complete response | 2 (1%) |
| - Partial response | 17 (7%) |
| - Progression | 107 (47%) |
| - Stabiliy | 103 (45%) |
| - Missing | 22 |
| 2^nd^ line Chemotherapy Type |  |
| - 1) Fluoropyrimidine-based | 92 (37%) |
| - 2) Gemcitabine combinations | 52 (21%) |
| - 3) Gemcitabine | 61 (24%) |
| - 4) Erlotinib - based | 1 (0%) |
| - 5) Other | 45 (18%) |
| Sequence of 1^st^ and 2^nd^ line chemotherapies  L1=FU based and L2=Gem combinations  L1=Gem combinations and L2=FU based | 48  21 |
| L1=Gemcitabine and L2= FU based | 15 |
| Sequence=Other | 167 |
| *Including: L1=FU based and L2=FU based* | *47* |
| *L1=FU based and L2=Gem combinations* | *58* |
|  |  |
|  |  |
